# Metabolic route computation in organism communities

**DOI:** 10.1186/s40168-019-0706-6

**Published:** 2019-06-07

**Authors:** Markus Krummenacker, Mario Latendresse, Peter D Karp

**Affiliations:** SRI International, 333 Ravenswood Ave., Menlo Park, 94025 CA USA

**Keywords:** Route search, Metabolic network, Microbiome, BioCyc, Pathway tools

## Abstract

**Background:**

Microbiomes are complex aggregates of organisms, each of which has its own extensive metabolic network. A variety of metabolites are exchanged between the microbes. The challenge we address is understanding the overall metabolic capabilities of a microbiome: through what series of metabolic transformations can a microbiome convert a starting compound to an ending compound?

**Results:**

We developed an efficient software tool to search for metabolic routes that include metabolic reactions from multiple organisms. The metabolic network for each organism is obtained from BioCyc, where the network was inferred from the annotated genome. The tool searches for optimal metabolic routes that minimize the number of reactions in each route, maximize the number of atoms conserved between the starting and ending compounds, and minimize the number of organism switches. The tool pre-computes the reaction sets found in each organism from BioCyc to facilitate fast computation of the reactions defined in a researcher-specified organism set. The generated routes are depicted graphically, and for each reaction in a route, the tool lists the organisms that can catalyze that reaction.

We present solutions for three route-finding problems in the human gut microbiome: (1) production of indoxyl sulfate, (2) production of trimethylamine N-oxide (TMAO), and (3) synthesis and degradation of autoinducers. The optimal routes computed by our multi-organism route-search (MORS) tool for indoxyl sulfate and TMAO were the same as routes reported in the literature.

**Conclusions:**

Our tool quickly found plausible routes for the discussed multi-organism route-finding problems. The routes shed light on how diverse organisms cooperate to perform multi-step metabolic transformations. Our tool enables scientists to consider multiple alternative routes and identifies the organisms responsible for each reaction.

**Electronic supplementary material:**

The online version of this article (10.1186/s40168-019-0706-6) contains supplementary material, which is available to authorized users.

## Background

Microbiomes harbor a multitude of different microbes that are living together in close contact. These microbes are interacting with each other synergistically, competitively, and antagonistically, by various mechanisms. One key interaction is the exchange of metabolites. To understand the functional capabilities and dynamics of a microbiome, knowing how exchanged metabolites hold together the microbiome’s overall metabolic network is necessary.

For example, indoxyl sulfate is derived from the breakdown of L-tryptophan by colon microbes, involving also the human host. It is an extensively studied uremic solute in humans, which has been implicated in toxicity among patients with kidney disease [1]. Clearance by hemodialysis appears to be limited, because indoxyl sulfate is mostly protein bound and shows limited diffusion across hemodialysis membranes. One problem is identifying the microbes in the human gut that participate in the synthesis of this toxic metabolite, and via which reactions and enzymes. Such results could potentially lead to clinical interventions. The sheer number of microbes and reactions potentially involved presents challenges for identifying relevant targets.

We have developed a software tool called *Multi Organism Route Search* (*MORS*) to propose plausible biosynthetic routes between researcher-supplied starting and ending (goal) metabolites, where such routes can span multiple microbes and other organisms, including the human host. MORS finds linear reaction sequences that convert the specified start metabolite to the specified final metabolite. Such a series of consecutive biosynthetic reactions is called a *route*. A key use case for MORS is exploratory searching for implicated organisms and reactions, when a goal metabolite is given, which may have been found in a metabolomics experiment.

## Implementation

During more than two decades, we have developed the BioCyc website [2] and its underlying software called Pathway Tools [3]. In the latest release 22.6, BioCyc publishes 14,560 metabolic organism databases, mostly bacterial. Additionally, 9 pan-genome databases are provided. Most databases were computationally generated; approximately two dozen received varying levels of expert human curation.

We introduced a single-organism Metabolic Route Search tool (called RouteSearch) in 2014 [4]. RouteSearch is designed to find optimal metabolic routes, according to a set of criteria, which a researcher interactively specifies and explores. Generally, longer routes are considered less optimal. Another optimality criterion is to retain as many atoms as possible from the start metabolite, such that they still are present in the goal metabolite. Inferring the retained atoms is enabled by pre-computed atom-mappings [5] between the metabolites of the reactions that are obtained from MetaCyc.

MORS extends the single-organism RouteSearch to enable route searches that utilize reactions from an arbitrary number of organism databases in the BioCyc collection. Thus, one new feature is to enable the user to select a set of organisms to consider in a particular search. An organism set can be selected in several ways: by searching for individual organism by name, by browsing alphabetical lists of organism names, by selecting organisms from the NCBI taxonomy [6], and by selecting organisms based on metadata recorded by the genome-sequencing project, which can include the Human Microbiome Project [7] (HMP) defined body site, in which the organism is found. Another new feature is the minimization of organism switches needed for completing a route, as described below.

To make MORS practical, finding an efficient way to perform route searches across an arbitrary subset of the 14,560 organism databases in BioCyc was essential. Our solution exploits the special role that our MetaCyc [2] database plays. MetaCyc is our master database that aims to cover the universe of chemical reactions that are present in our BioCyc collection. After expert curators record reactions in MetaCyc, based on published experiments, MetaCyc serves as the master template to predict the reactions and pathways in other BioCyc organism databases by the PathoLogic algorithm [8]. Therefore, the vast majority of reactions within BioCyc databases are also present in MetaCyc, and the same unique identifier assigned to a reaction *R* in MetaCyc is assigned to *R* in every other BioCyc database in which it occurs.

The exceptions are transport reactions that are inferred by our Transport Inference Parser [9] based on gene annotations, which can create novel reactions not in MetaCyc. Additionally, some manually curated organism databases contain new reactions that are not present in MetaCyc. To accommodate these differences, we constructed a new database called MetaRoute, into which we first copied all the metabolic reactions from MetaCyc. Then, we imported into MetaRoute the extra reactions that were present in other organism databases but not in MetaCyc. Thus, MetaRoute spans all metabolic reactions found in BioCyc. However, MetaRoute lacks transport reactions and MORS does not currently use transport reactions, because most genome annotations fail to annotate the substrates of significant numbers of transporters. If transport were required by MORS whenever adjacent reactions were catalyzed by different organisms, then generation of many valid routes would be prevented. Furthermore, MORS operates in a compartment-agnostic manner, meaning reactions are not segregated into separate compartments. Unsegregated metabolites have been used before in multi-organism investigations, e.g., [10, 11].

To speed the execution of MORS, we pre-compute a 2D binary array whose rows are reaction IDs in MetaRoute and whose columns are BioCyc organism IDs. At the intersection of a particular reaction ID and a particular organism ID, the bit is either set if this reaction is in that organism or unset otherwise. This array enables quickly determining all the reactions that a given organism in BioCyc contains, without even having to open and load that particular database. Constructing this array requires opening all BioCyc organism databases and takes several hours of processing time. A separate, pre-computed database contains genomic metadata for each BioCyc organism, so we can rapidly obtain the list of organisms for a specific HMP body site, for example. Combined, these pre-computed data enable efficiently finding the union of all reaction IDs expected for the set of organisms the researcher has selected.

MORS provides an additional property that is minimized during route searches, in addition to the RouteSearch costs of lost atoms and of the number of reactions in the route. MORS also minimizes switching of organisms in a route. A switching of organisms occurs in a route when a reaction *R*_1_ leads to a reaction *R*_2_ where the set of organisms containing *R*_1_ shares no organism with the set of organisms containing *R*_2_. In other words, the sets of organisms containing *R*_1_ and *R*_2_ have an empty intersection^1^. We minimize organism switching on the assumption that each such switch could render a route inoperable if the organisms catalyzing adjacent reactions do not have the ability to export and import, respectively, the substrate shared between those adjacent reactions. Since each of the minimized properties (atoms lost, reaction length, and organism switches) has a user-controlled weight, the user can adjust their their relative contributions to overall route cost.

The search algorithm was modified to minimize organism switching given a user-provided cost for one switching of organisms. The costs of all organisms switches are added to the other costs, such as the cost of lost atoms, in evaluating a route. As before, the overall minimum cost route is considered the best route. The algorithm to detect switching of organisms maintains a set *S* of organisms as it proceeds to find a route from the source compound to the target compound. The initial value of *S* is the subset of organisms, from the user-specified set of organisms, which contains the first reaction of the route. *S* is updated when expanding the route with a reaction *R* by the intersection of *S* and the set of organisms containing *R*. Switching of organisms in a route is detected when *S* becomes empty, at which point, the set of organisms *S* is reinitialized to the user-specified set of organisms that contains the most recently added reaction *R*, and the user-selected cost of switching is added to the cost of the route. Note that during the search of a route, *S* is a set of organisms that contains all the preceding reactions up to the last switching. That algorithm computes not only the cost of switching, but also a series of maximal sets of organisms that maintains a minimum switching strategy^2^.

Indeed, as just discussed, for each reaction *R* in the route, a set *S*_*R*_ is computed based on the intersection operation, which is the maximal set of organisms that we can have, to not force a switching to other organisms. Note that maximal means that no organism can be added to that set, such that this organism would contain all the reactions up to the last switching of organisms. Naturally, if the set *S*_*R*_ becomes empty, a switching occurs and that set is reinitialized to the set of organisms that contains *R*, which, by definition, is also the maximal set of organisms that contain *R*. By induction, the maximal property is true for all sets *S*_*R*_ of the route.

For efficiency, the intersection of organism sets is implemented by bit-vector operations. We have considered using a sparse representation of sets when the user selected a large set of organisms (> 200), but that does not appear useful, because many reactions belong to many different organisms resulting in large non-sparse bit vectors.

Commonly, single start and goal metabolites will be specified. But consider that in some cases, the start (or goal) metabolite may be unknown. In a case where the start metabolite is not known, the researcher may want to direct the tool to explore routes from any of a set of start metabolites to determine which is the most plausible candidate. With an additional set of selectors, the researcher can specify a starting metabolite set, or a goal metabolite set, as the set of metabolites that are recorded in a BioCyc SmartTable. A SmartTable is a stored collection of BioCyc objects [12]. SmartTables have to be created beforehand, by one of the numerous methods available. The metabolites have to be placed into the first (leftmost) column of the SmartTable. For more on SmartTables, please see https://biocyc.org/PToolsWebsiteHowto.shtml#smarttables.

For convenience, we have pre-defined a SmartTable containing a useful set of start or goal metabolites consisting of 13 common intermediate metabolites of central metabolism. This SmartTable can be chosen within the MORS input dialog, and its metabolites are listed in Table 1. The rationale for the chosen metabolites was that they frequently serve as starting or ending points for many biosynthetic and degradative pathways.

**Table 1:** These 13 metabolites are common intermediates of central metabolism

Metabolite	MetaCyc ID	No. of pathways
*β*-D-fructofuranose 6-phosphate	FRUCTOSE-6P	75
2-Oxoglutarate	2-KETOGLUTARATE	354
3-Phospho-D-glycerate	G3P	45
D-Erythrose 4-phosphate	ERYTHROSE-4P	31
D-Glucopyranose 6-phosphate	D-glucopyranose-6-phosphate	74
D-Glyceraldehyde 3-phosphate	GAP	81
D-Ribofuranose 5-phosphate	CPD-15317	12
D-Sedoheptulose 7-phosphate	D-SEDOHEPTULOSE-7-P	19
Acetyl-CoA	ACETYL-COA	369
Oxaloacetate	OXALACETIC_ACID	66
Phospho enol pyruvate	PHOSPHO-ENOL-PYRUVATE	83
Pyruvate	PYRUVATE	336
Succinyl-CoA	SUC-COA	77

The last column indicates the count of MetaCyc pathways involving the metabolite

The researcher can also specify the number of routes to compute, the maximum allowed time for the computation, and the maximum route length. Longer routes can take substantially more time to find, because a deeper tree of choices must be traversed, which, in the worst case, could lead to an exponential time increase. Therefore, a search may possibly time out, before truly optimal routes may have been discovered, based on the search criteria entered. In this case, it might be best to experiment with increasing the allotted time or changing start and/or goal metabolites to similar candidates that could lead to shorter routes.

MORS presents computed routes at the bottom of the results page as SVG graphics, sorted by increasing computed cost. Each route is a linear succession of reactions, connecting a start to a goal metabolite and showing the intermediates. Mouse-over tooltips on reaction arrows show the full reaction equation and state the count of the organisms that contain the reaction. If there are few organisms, they will be listed by name. The full table of organisms can be captured in a SmartTable and subsequently exported.

## Results

The following results were obtained with version 22.6 of BioCyc. The size of the pre-computed binary reaction array is 17,342 reactions versus 14,562 organisms.

Table 2 shows a listing of the catalytic capabilities of the microbial communities within the nine HMP body sites that are currently recorded in our organism metadata. The table shows the counts for the organisms at each body site (one BioCyc database per organism), and the number of reactions that can be catalyzed by the organisms within each body site. Each reaction count is computed as the union across all organisms in the body site of the reactions catalyzed by that organism. The procedures used to construct BioCyc databases ensure that the same biochemical reaction always receives the same unique identifier in a new database; therefore, we compute the union of the reactions within two databases by computing the union of the BioCyc unique identifiers of those reaction sets.

**Table 2:** In BioCyc 22.6, the available HMP body sites contain these tabulated numbers of microbes and reactions

Body site	Organisms	Reactions
Airways	99	4059
Blood	281	4681
Bone	10	2818
Central nervous system	25	2859
Ear	2	1141
Eye	1	1173
Gastrointestinal tract	674	5105
Heart	3	1680
Liver	1	925
Lymph nodes	2	1645
Nose	4	2319
Oral	408	5102
Skin	171	3980
Urogenital tract	455	4897
Wound	21	3125

For the entire table, the set union of all the organisms amounts to 2135, and the set union of all the reactions amounts to 6006.

Below, we show several example routes computed by MORS. For all examples, the same set of organisms was used, namely all organisms in the human microbiome body site called “gastrointestinal-tract” plus *Homo sapiens*. In total, this amounted to 675 organisms, which we call the GI-Human set. The organism count refers to individual strains. We like the GI-Human set, because it is one of the most studied microbiomes, such that example routes can be found that also have been described in the literature, to verify the results produced by MORS.

The researcher invokes MORS by the BioCyc website menu command **Metabolism →Metabolic Route Search** and then clicking on the checkbox next to “Routes across Multiple Organisms?”. Please see Additional file 1 for a step by step walkthrough of running example 1. Many of the parameter settings are shared between the examples. All parameters not explicitly mentioned were left at their default values. The computational times for finding the routes in the examples are summarized in Table 3, to illustrate that when routes are long, they can consume substantially more time when minimization of organism switching is enabled. In most examples, we set the “switching organisms cost” parameter at its default value of 30. We recorded the organisms catalyzing each reaction for the example routes as spreadsheets in Additional file 2.

**Table 3:** A comparison of the times to find routes

Example route	No. of routes	Max. route length	Time (no OSM)	Time (with OSM)
L-Tryptophan →indoxyl sulfate	3	9	25 s	161 s
L-carnitine →TMAO	3	7	9 s	115 s
SAM →L-homoserine lactone	4	4	9 s	9 s

Each example route search was run without or with organism switching minimization (OSW). OSW is turned off when the “switching organisms cost” parameter is set to zero. This parameter was set to 30 for the runs with OSW. Because the run time can be very sensitive regarding the “maximum route length” parameter, these settings are also shown in the table. The data was collected with MORS running on a single CPU core of type Intel(R) Xeon(R) CPU E5-2690 0 @ 2.90 GHz.

### Example 1: L-tryptophan to indoxyl sulfate

Indoxyl sulfate is implicated in toxicity among patients with kidney disease [1]. Dietary L-tryptophan is converted to indoxyl sulfate through a known route of three reactions in which L-tryptophan is first degraded to indole by gut microbes [13]. After indole enters the bloodstream, the liver applies two further modifications, resulting in indoxyl sulfate [14].

To see whether MORS can find this route, we selected the GI-Human set described previously. The start compound was L-tryptophan and the goal compound was indoxyl sulfate. We set the “number of routes” to 3 and the “maximum route length” to 9.

**Fig. 1:**
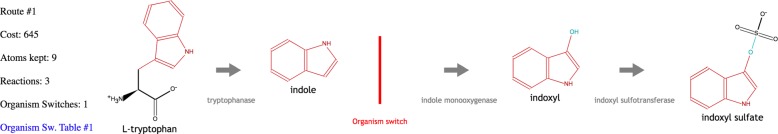
The optimal route from L-tryptophan to indoxyl sulfate. This route retains 9 atoms, over 3 reaction steps

MORS was found as the two top routes the expected route discussed above, consisting of three reaction steps, retaining nine atoms, shown in Fig. 1. The two top routes are very similar and differ only in the first reaction. These reactions differ in a side-product, which is not explicitly shown in the route. The first reaction can be catalyzed by 3 different microbes in the top route and by 125 different microbes in the second route. The last two reactions of the top routes are catalyzed by *Homo sapiens* only. The single organism switch is clearly indicated in the pathway diagram in Fig. 1.

A third route of nine reactions is also found, retaining seven atoms and involving one organism switch (see Additional file 3). Although this route appears biochemically possible, it looks less likely, because the route is much longer. The first seven steps, up to the organism switch, can be catalyzed by a set of 7 organisms, which are listed in Additional file 2.

### Example 2: L-carnitine to TMAO

Dietary L-carnitine from red meat is converted to TMAO in a known route of two reactions in which L-carnitine is first degraded to trimethylamine (TMA) by gut microbes; the liver further converts TMA to trimethylamine N-oxide (TMAO) [15]. TMAO is implicated in accelerating atherosclerosis [15].

To see whether MORS can find this known route, we again began with the GI-Human organism set. The start compound was L-carnitine and the goal compound was TMAO. We set the “number of routes” to 3 and the “maximum route length” to 7.

**Fig. 2:**
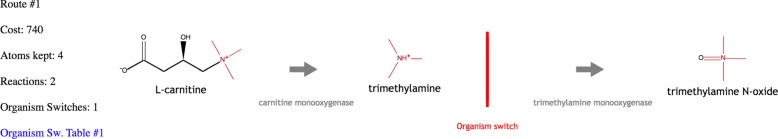
The optimal route from L-carnitine to TMAO. This route retains 4 atoms, over 2 reaction steps

MORS found the expected route, discussed above, as the top route, consisting of two reaction steps, retaining four atoms, shown in Fig. 2. The first reaction can be catalyzed by 47 different microbes, whereas the last reaction is catalyzed by *Homo sapiens* only. The single organism switch is depicted in the figure.

A second and third route, both containing six reactions, are also found. Both retain four atoms and involve one organism switch (see Additional file 4). The routes are very similar, differing in their first reaction only. The first four steps of both routes convert L-carnitine to *γ*-butyrobetaine, which then is converted to TMA by a reaction that is very similar to the first step in the known route. Thereafter, TMA is converted to TMAO by the same reaction step as in the known route. This last reaction is catalyzed by *Homo sapiens* only, whereas the second to last is catalyzed by 44 different microbes. Therefore, the first five steps all were found to be catalyzable by at least 44 microbes. The routes differ in their first step, which utilizes a carnitine-CoA ligase in the second route, whereas it is a *γ*-butyrobetaine-CoA:carnitine CoA transferase in the third route. An interesting paper made the case that dietary L-carnitine is first primarily converted to *γ*-butyrobetaine, at a rate three orders of magnitude higher than the formation of TMA [16]. Thereafter, *γ*-butyrobetaine is converted to TMA in a downstream part of the gut, by a somewhat different microbial community. MORS was able to explore such longer and apparently more physiologically relevant routes, despite their higher cost.

### Example 3: Autoinducer degradation

Autoinducers are compounds involved in quorum sensing, which is a microbial communication mechanism. In a microbiome, where many different types of microbes live closely together, finding evidence of different microbial species influencing each other, by means of signaling molecules, may be possible. We wanted to see whether MORS could be used for examining the boundary between the organisms that produce particular autoinducers and those that degrade them.

By browsing autoinducer metabolism in MetaCyc, we saw that L-homoserine lactone was a degradation product common to a class of autoinducers called acyl-homoserine lactones (AHLs). So we picked L-homoserine lactone as the goal compound. As the start compound, we chose S-adenosyl-L-methionine (SAM), the known source for the biosynthesis of this class of autoinducers.

We again selected the GI-Human organism set. We set the “number of routes” to 4 and the “maximum route length” to 4, because a large value such as 10 led to a time-out. One possible cause could be that S-adenosyl-L-methionine is used in a very large number of reactions, so the search tree is very large and time consuming to traverse.

**Fig. 3:**
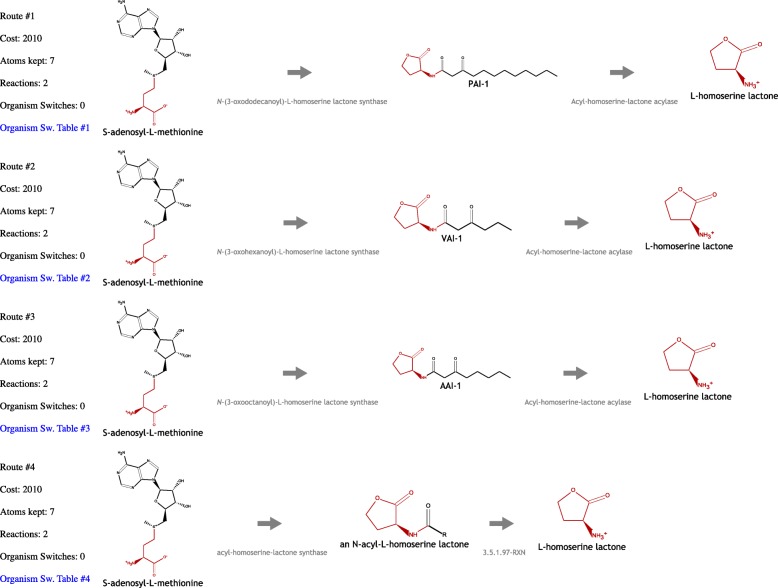
Four routes connecting autoinducer synthesis and degradation. All four routes follow a similar pattern of 2 reaction steps and retaining 7 atoms, which originate from a fragment of S-adenosyl-L-methionine. The differences arise from the lipid side chain of the particular autoinducer involved

MORS found several related routes, which all have two reactions, retaining seven atoms and involving zero organism switches, shown in Fig. 3. The differences between the routes is that the actual autoinducers (the AHLs) are different, which are the products of the first reaction steps. The first reactions are catalyzed by six organisms, whereas the second reaction steps are catalyzed only by one organism, namely Pseudomonas aeruginosa 2_1_26. No organism switching was needed to complete these routes because this organism also occurs in the first set of five organisms. So in principle, this organism can perform the entire transformation on its own.

However, by setting the “switching organisms cost” parameter to 0 (zero) and re-running the search, a slightly different result is obtained. One microbe from the set of organisms for the second step, *Ralstonia pickettii 5_7_47FAA*, does not occur in the organism set for the first reaction, indicating an example of a microbe that solely degrades a signaling compound synthesized by other microbes, which could lead to an asymmetrical interference with quorum sensing.

## Discussion

The complexities of organism diversity in microbiomes and of the metabolic networks in each of these organisms present challenges to our understanding of what exactly are the metabolic capabilities of a microbiome. We have developed an extension of our RouteSearch tool [4], such that routes can be found that traverse researcher-defined sets of organisms. Additionally, MORS can minimize the number of organism switches in a route. As we have shown, MORS finds routes to metabolites that may affect human health and which have been experimentally shown to originate from microbial activity.

The accuracy of MORS predictions depends on several factors. One is the quality of the metabolic reconstructions in BioCyc. The vast majority of organism databases in BioCyc have been computationally predicted from annotated genomes; the quality of the resulting databases depends on the thoroughness and correctness of the original genome annotation. Their quality also depends on the state of curation in MetaCyc, our master database that is used as a template for metabolic reconstruction. Additionally, the reconstructions depend on the correctness of the PathoLogic reactome prediction algorithm [8].

Cellular compartments are currently not taken into account by MORS. All metabolic reactions among the selected organisms are considered by the search to be fully accessible. In an actual collection of microbes, cell compartments will segregate many reactions, which will thus be unavailable, unless specific transporters are also added to the metabolic network. Generally, it appears that correctly predicting transport reactions computationally is difficult. The compartment-agnostic operation of MORS reduces false negative predictions at the expense of increasing false positives, which seems the better trade off for exploration. However, extending MORS to optionally take transporters and compartments into account could increase the realism of the routes found.

## Conclusions

We developed a software tool called MORS, which efficiently finds plausible metabolic routes within researcher-specified subsets of the BioCyc collection of 14,560 organism databases. MORS searches for optimal metabolic routes that minimize the number of reactions in each route and maximize the number of atoms conserved between the starting compound and the ending compound. Multiple routes can be found after computing for a few seconds to a few minutes, depending on the details of the search. MORS pre-computes a bit array describing the presence of metabolic reactions in the BioCyc databases; that array facilitates fast computation of the full set of reactions present in a researcher-specified set of organisms. MORS calls the previously developed RouteSearch algorithm to search for optimal metabolic routes. MORS enables the researcher to search between individual start and goal metabolites. In addition, when the researcher is unsure of the start or goal metabolite, the researcher can specify a set of compounds for either the start or the goal.

We have demonstrated the utility of MORS by finding routes to several microbiome-related metabolites that affect human health, which have been previously reported in the experimental literature. In the first two cases, where routes were previously known in the literature, the optimal solution found by MORS matched the route in the literature. In our third example, MORS generated hypothetical routes for the synthesis and degradation of several autoinducer compounds.

For future work, we would like to work with experimental collaborators to generate routes to additional microbiome-relevant metabolites, to help determine the metabolic origins of these metabolites. Such collaborations could lead to implementing additional features and support for more complex and targeted route searches.

## Availability and requirements

Project name: Multi Organism Route Search (MORS)Project home page: https://biocyc.org/ via the menu commandMetabolism →Metabolic Route Search.Operating systems: MacOS, Windows, Linux.Programming languages: Common LISP, JavaScript, SVG. The MORS source code is freely available to academic institutions upon request, as part of version 22. 5 (September 2018) of the Pathway Tools software.Other requirements: A modern Web browser is recommended. MORS needs SVG graphics, which does not work correctly on Safari, Chrome, and Internet Explorer. Please choose Firefox as the browser. We tested on Firefox 56.0.2 on MacOS High Sierra (10.13.6) and on Firefox 65.0.1 on MacOS 10.11.6. Because of the problems with SVG, we are looking into changing the display code to use HTML 5 technology, instead of SVG. Once this is done, hopefully the full range of modern browsers will be supported in a future BioCyc release.License: Access to organism databases in BioCyc, other than MetaCyc and EcoCyc, requires a subscription. However, BioCyc provides a level of free accesses per month to each researcher. The results reported here were obtained using version 22.6 of BioCyc. Subsequent versions of BioCyc are likely to show differing numbers for organism sets and may show variations in the exact routes found. The route searching directly depends on the available data in our collection of PGDBs, which over time increases in size, amount of curation, and automated inferences that we might apply.

## Additional files

Additional file 1 Walkthrough of the MORS User Interface. We provide a step by step guide, with screen snapshots, for setting up the parameters in the MORS Graphical User Interface (GUI), to run the route search for example 1 from the main paper. The steps easily generalize to the other examples. (PDF 2722 kb)

Additional file 2 Excel worksheet of organism tables for example routes. This Excel workbook of spreadsheets provides organism tables for several example routes, which were exported from the corresponding SmartTables. Such tables describe the organisms that can catalyze each reaction step in a MORS route. The format of the tables is described in Additional file 1, on the last page. For example 1, three routes are included, and for example 2, three routes are included. These routes are all for when organism switching minimization is used. For example 3, route 1 is included, once with and once without organism switching. Together, eight spreadsheets are provided, and the file was saved in the Microsoft Excel 5.0/95 Workbook format. (XLS 125 kb)

Additional file 3 L-tryptophan to Indoxyl Sulfate, two routes. The MORS interface controls and their selections are shown, followed by the resulting two routes computed by MORS. (PNG 756 kb)

Additional file 4 L-carnitine to TMAO, three routes. The MORS interface controls and their selections are shown, followed by the resulting three routes computed by MORS. (PNG 594 kb)

## Footnotes

1 The set of organisms containing a reaction depends on the user-selected set of organisms for the current MORS search.

2 There might be more than one series of maximal sets of organisms with a minimum number of switching of organisms in a route, but this algorithm provides only one of these series.
