# Supplementary material for: Metabolic route computation in organism communities
Source: Microbiome. 2019 Jun 7;7:89. doi: 10.1186/s40168-019-0706-6 (PMC6556054; doi:10.1186/s40168-019-0706-6)
Supplement: Supplementary file 1 — Walkthrough of the MORS User Interface. We provide a step by step guide, with screen snapshots, for setting up the parameters in the MORS Graphical User Interface (GUI), to run the route search for example 1 from the main paper. The steps easily generalize to the other examples. (PDF 2722 kb) [file 40168_2019_706_MOESM1_ESM.pdf]

Walkthrough of the MORS User Interface

Here, we provide a step by step guide for setting up the parameters in the MORS Graphical User Interface (GUI), to run the route search for Example 1 from the main paper. Example 1 finds routes from L-tryptophan to indoxyl sulfate. Modifying a few of the steps below for searching routes that have different start and goal metabolites, or differing organism sets, should be straight forward.

Each figure captures one step in the procedure, and shows a screen snapshot from a Web browser that depicts the result of the operation that is described in the figure's legend.

A modern Web browser is recommended. MORS needs SVG graphics, which does not work correctly on Safari, Chrome, and Internet Explorer. Please choose Firefox as the browser. We tested on Firefox 56.0.2 on MacOS High Sierra (10.13.6) and on Firefox 65.0.1 on MacOS 10.11.6. Because of the problems with SVG, we are looking into changing the display code to use HTML 5 technology, instead of SVG. Once this is done, hopefully the full range of modern browsers will be supported in a future BioCyc release.

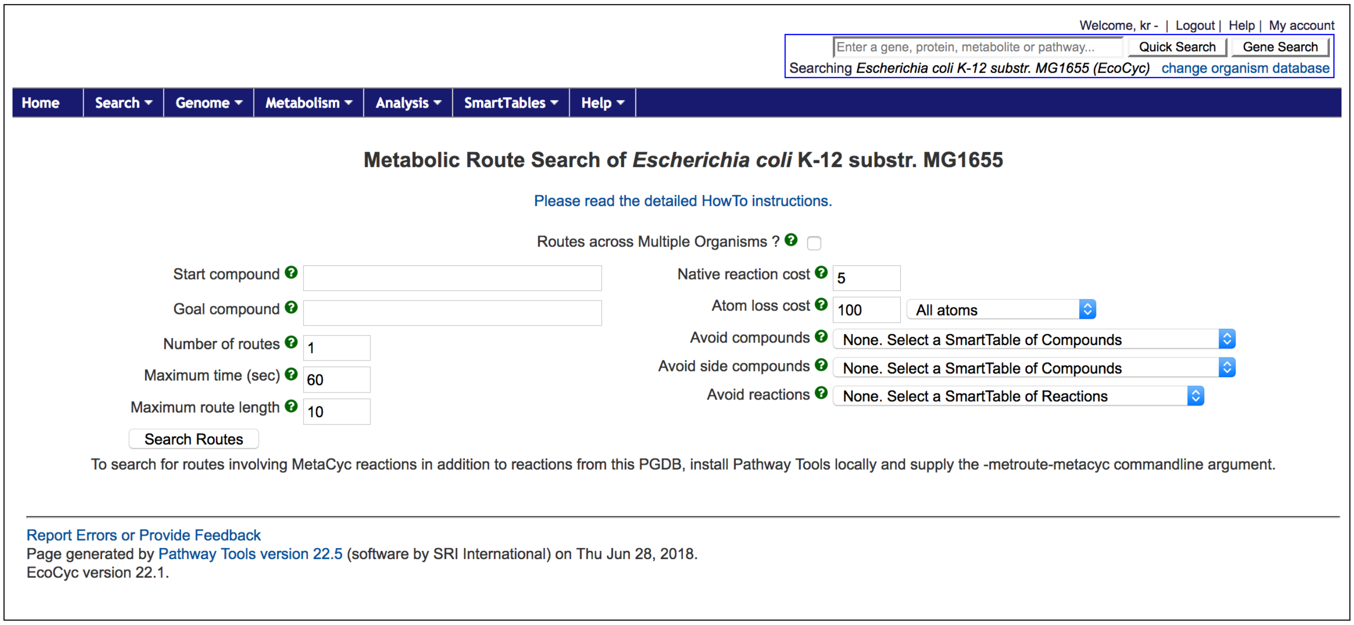

Figure 1: On BioCyc, accessible at `https://biocyc.org/`, select the menu command **Metabolism→Metabolic Route Search**, which then displays the Route Search page, which is an editable form for specifying the parameters of a search. Directly underneath the title is a link to additional detailed HowTo instructions, which should be read. Those instructions can also be consulted for discussions of parameters not mentioned in the walkthrough example here. Please also note that each parameter entry box has a green icon containing a question mark. Clicking on these help buttons will show specific help information in a small popup window.

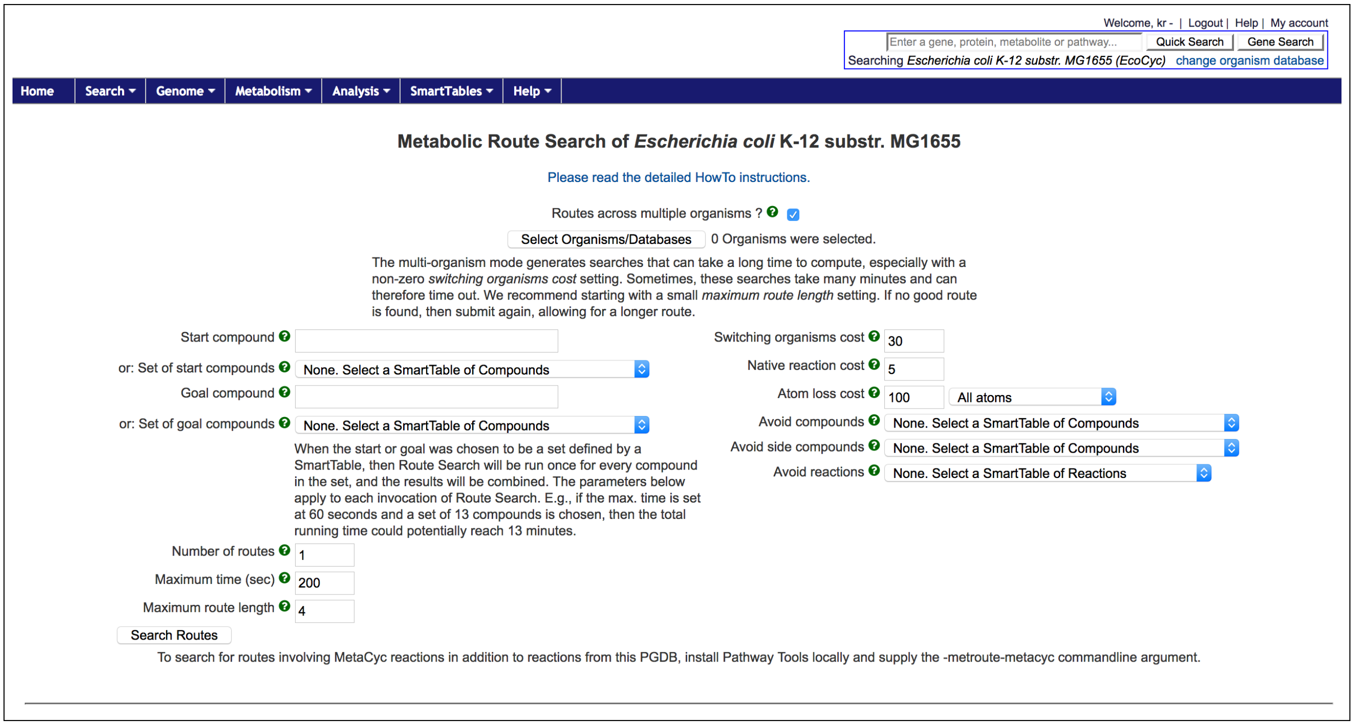

Figure 2: Select the MORS mode by clicking the checkbox called **Routes across Multiple Organisms ?**. Thereafter, additional GUI controls are exposed, incuding the **Select Organisms/Databases** button.

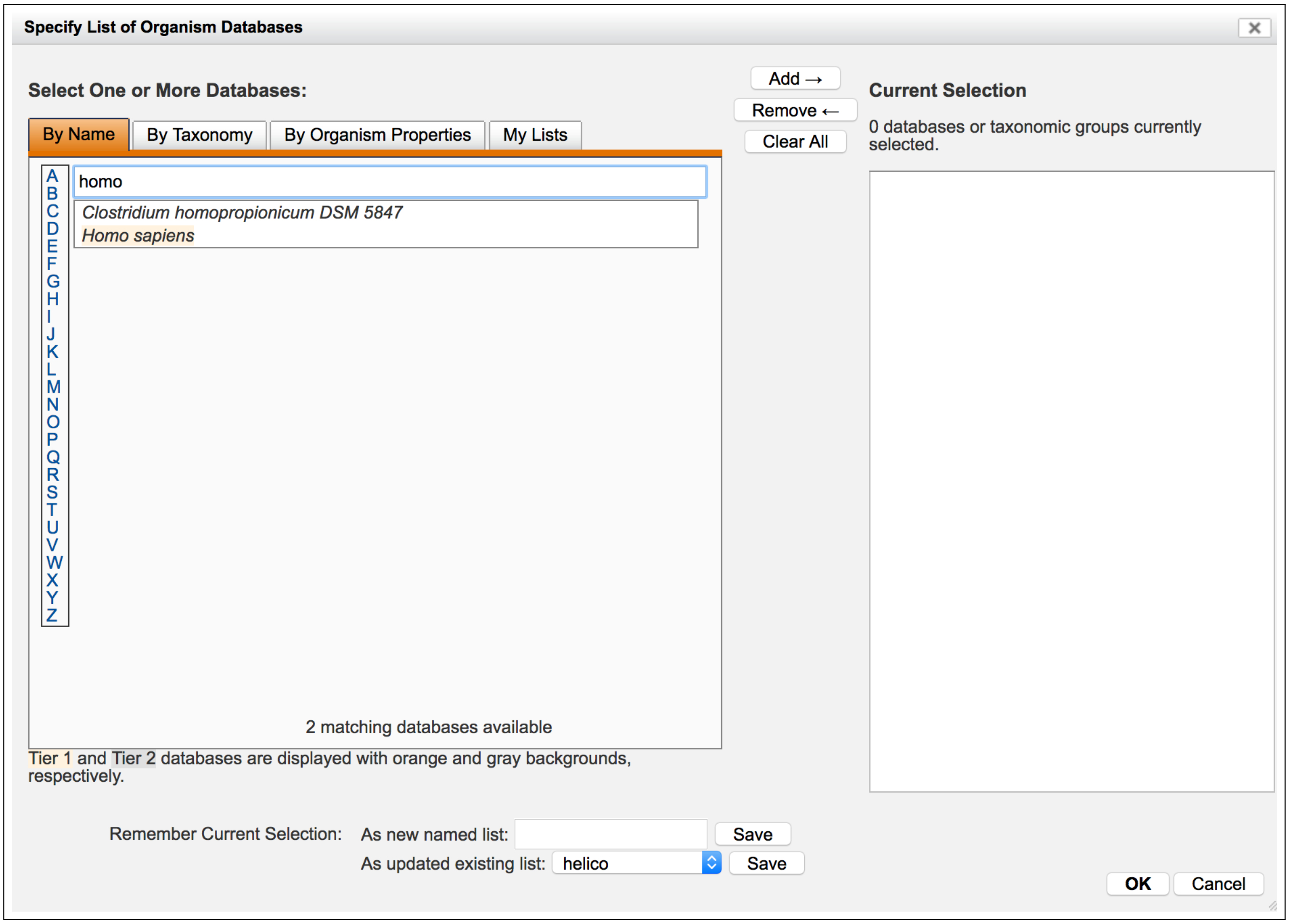

Figure 3: Click on the **Select Organisms/Databases** button to bring up this popup shown here, which is the selector titled **Specify List of Organism Databases**. First, choose the tab **By Name**, where typing a substring of an organism shows an increasingly narrowed choice of matching names. Click on “Homo sapiens” to select this organism and add it to the **Current Selection** column on the right hand side.

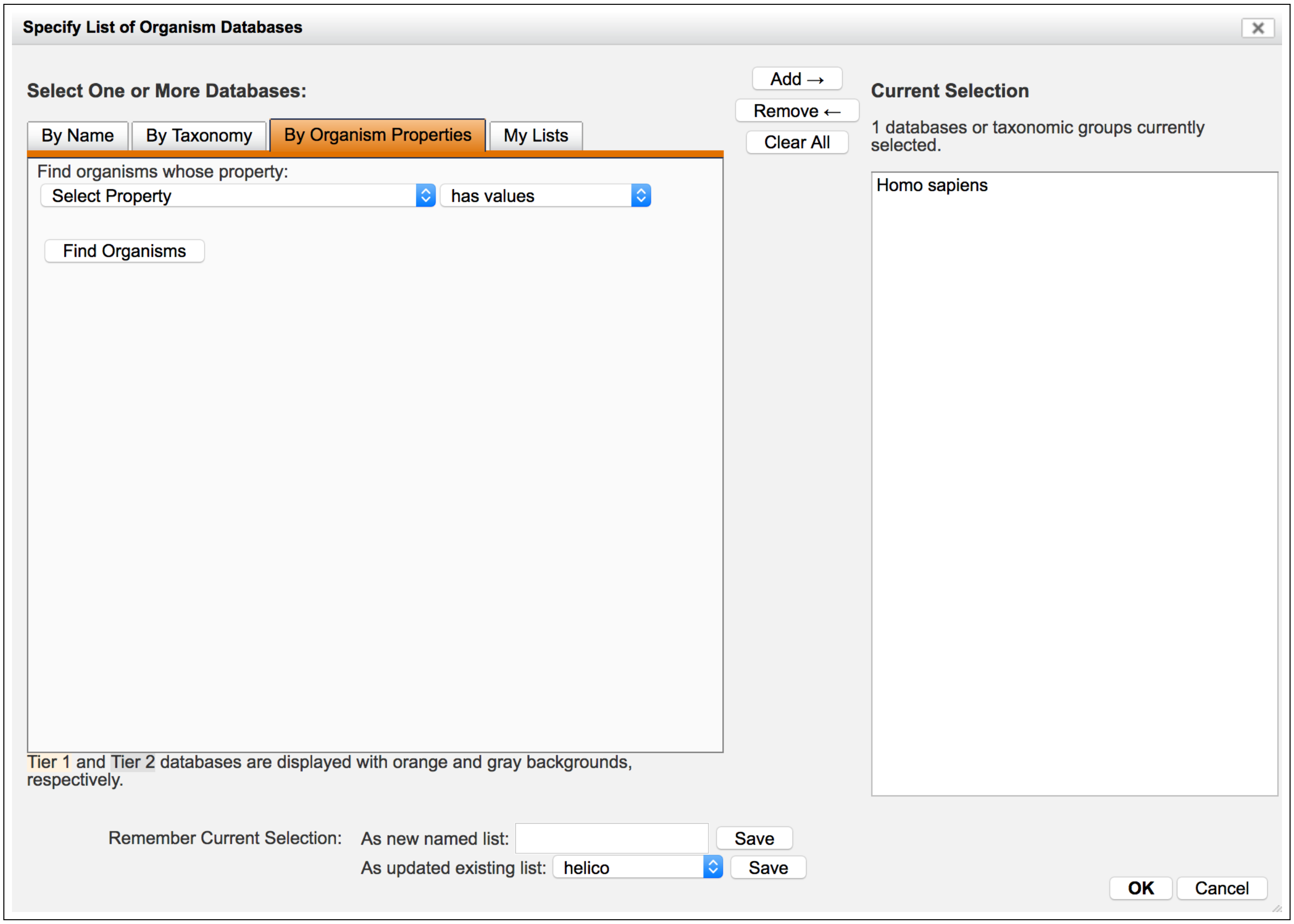

Figure 4: Second, choose the tab **By Organism Properties**. The additionally revealed controls enable selecting organisms based on metadata recorded by the genome-sequencing project.

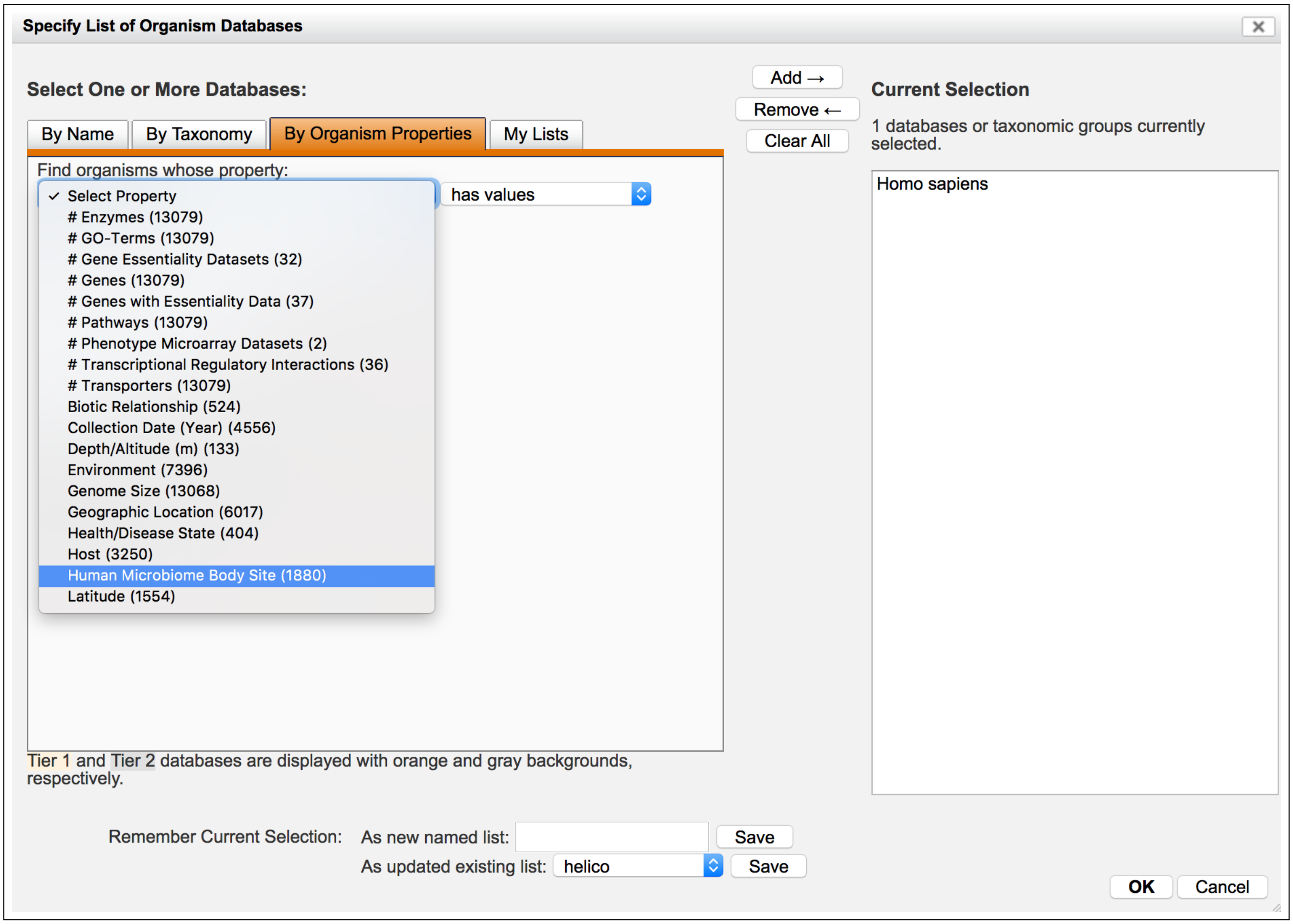

Figure 5: As the first step, click on **Select Properties** to list the provided choices. For this example, choose **Human Microbiome Body Site**. The number in parentheses states how many organisms were labelled with the respective property.

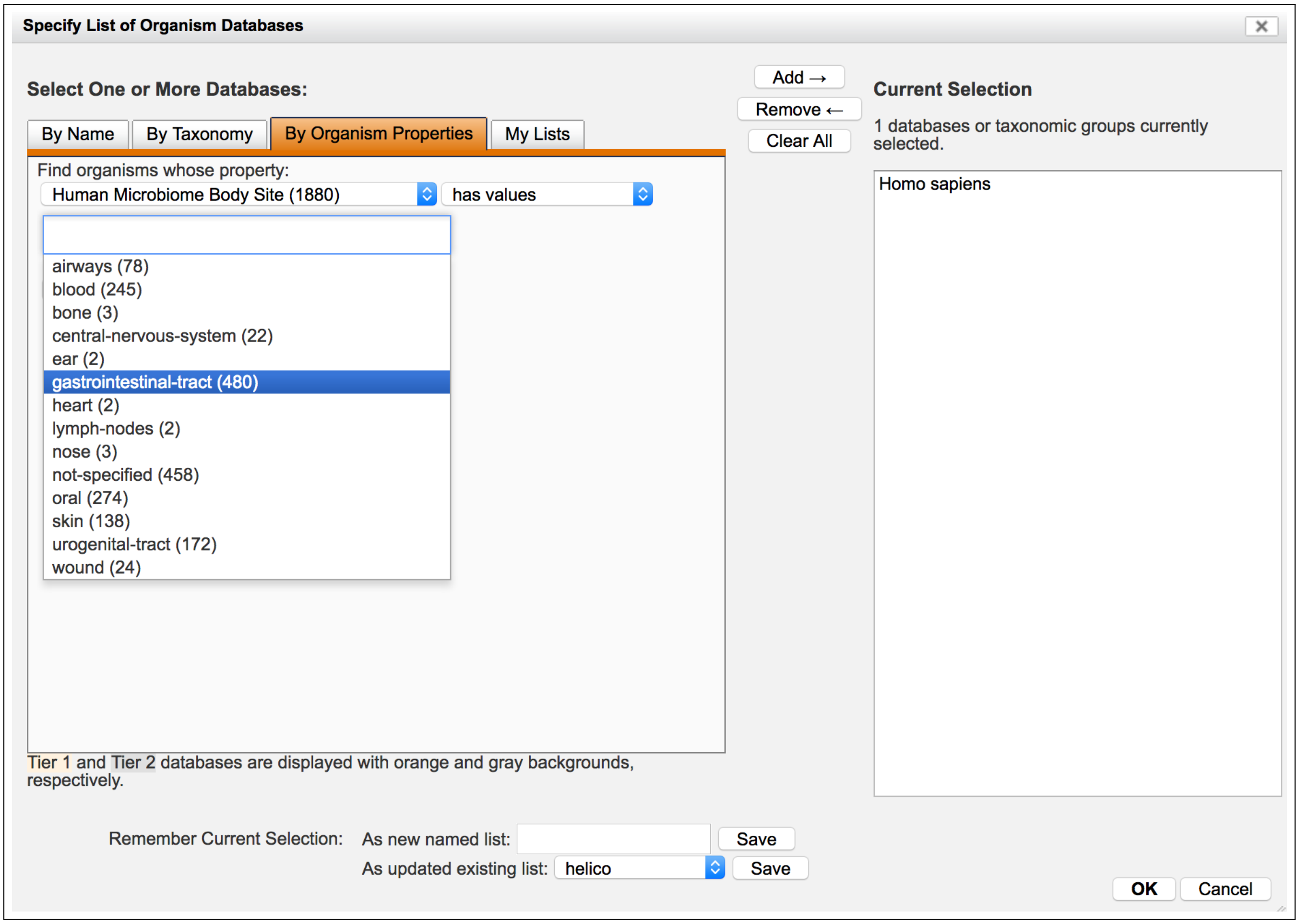

Figure 6: As the second step, click on the newly revealed selector that states **Click to see possible values**. For this example, choose **gastrointestinal-tract**. The number in parentheses again states how many organisms were labelled with the respective property.

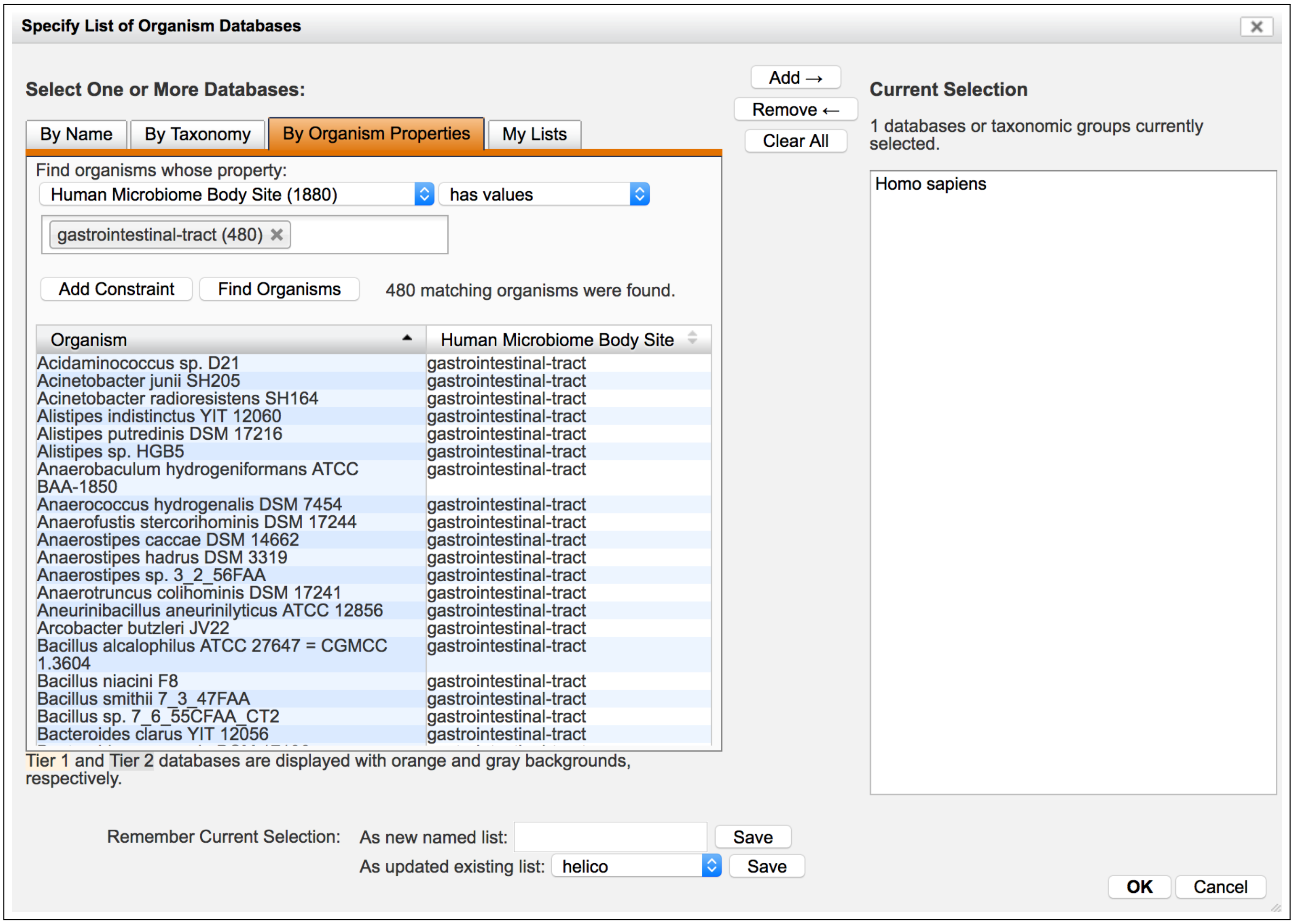

Figure 7: As the third step, click on the button labelled **Find Organisms**. As the result, all the organisms that fulfill the search criteria are listed underneath, to enable selecting a subset of organisms.

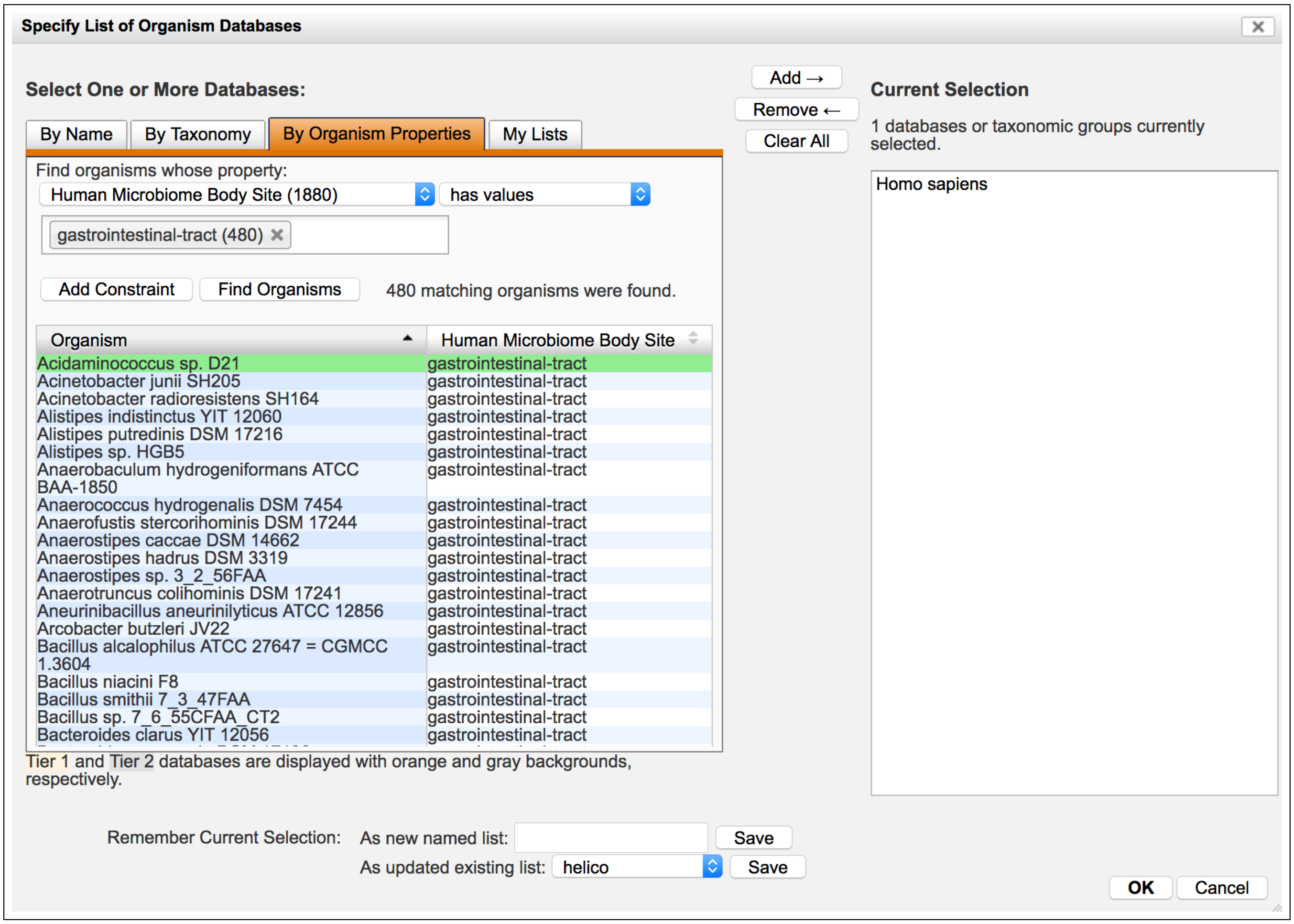

Figure 8: To select one organism, simply click on it, which marks it green, thereby indicating its selected status.

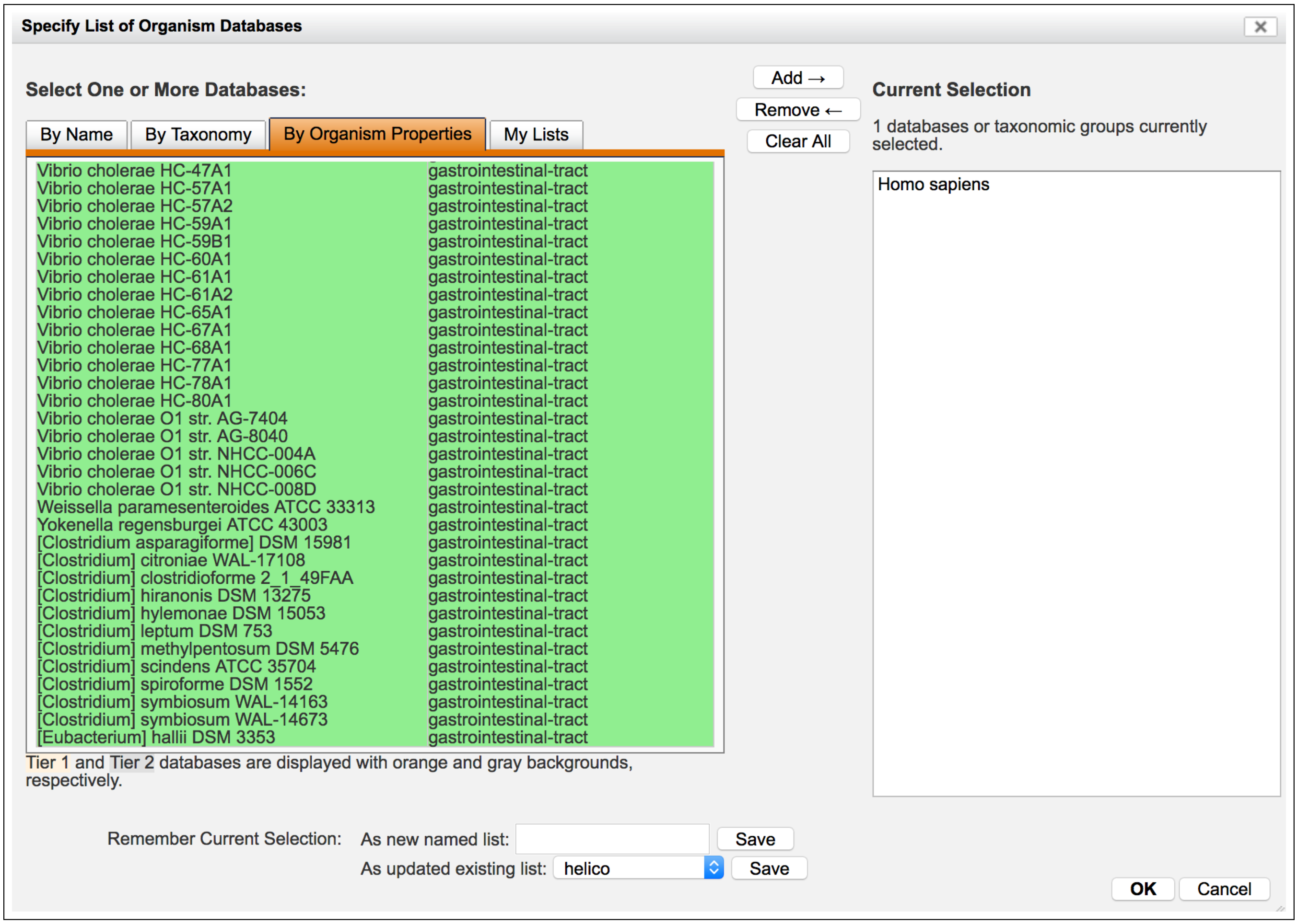

Figure 9: To select all organisms, select the first organism in the list (as just described above). Then, after scrolling all the way down to the end of the list, also select the last organism by holding down the shift key and clicking. This sequence of operations selects the entire range of organisms. Click on the **Add** → button to add this set of organisms to the **Current Selection** column on the right hand side. To finalize the selection and return the organism list to MORS, click on the **OK** button, which also closes the organism selector popup.

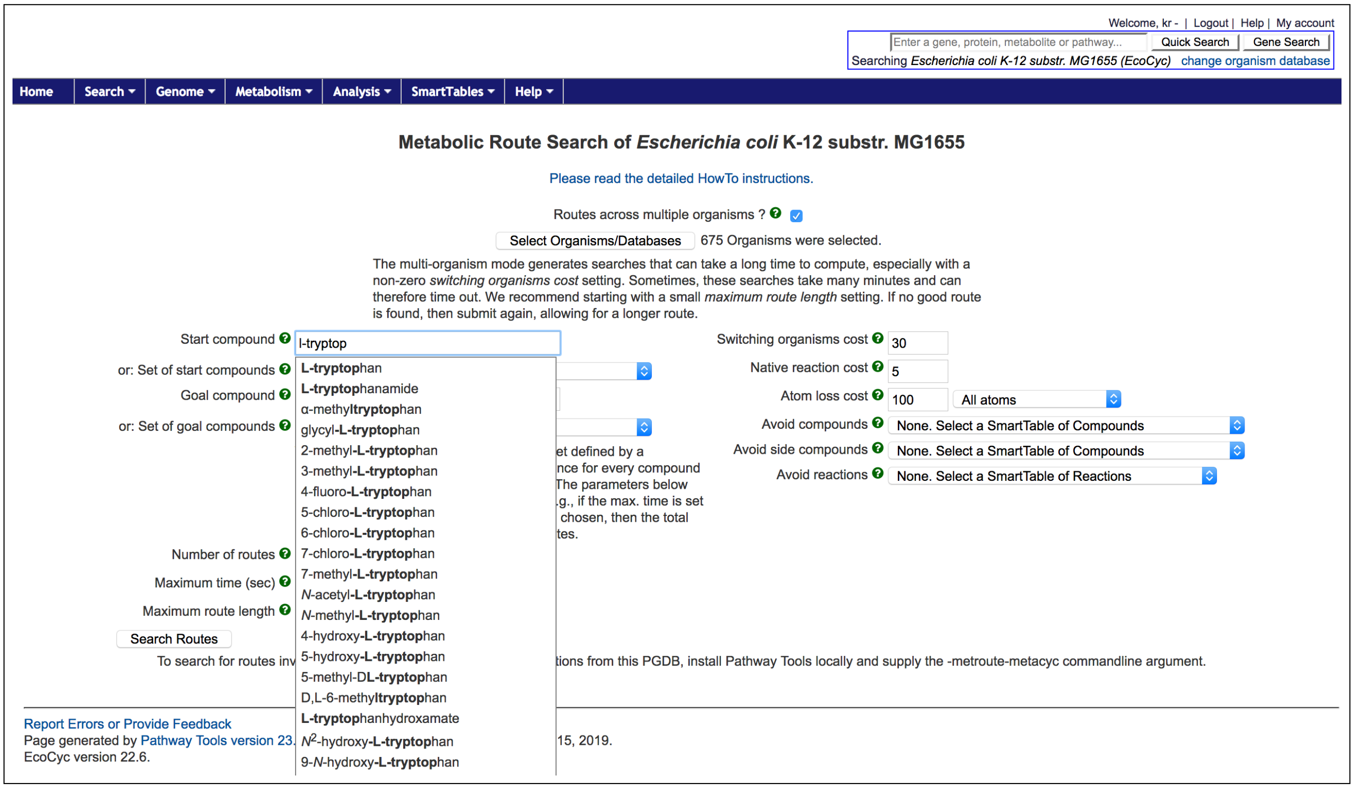

Figure 10: After the organism set has been selected, enter the start metabolite by typing a substring of the name in the text box, which causes a list of plausible completions to be shown underneath. After typing enough characters, select **L-tryptophan** from the list.

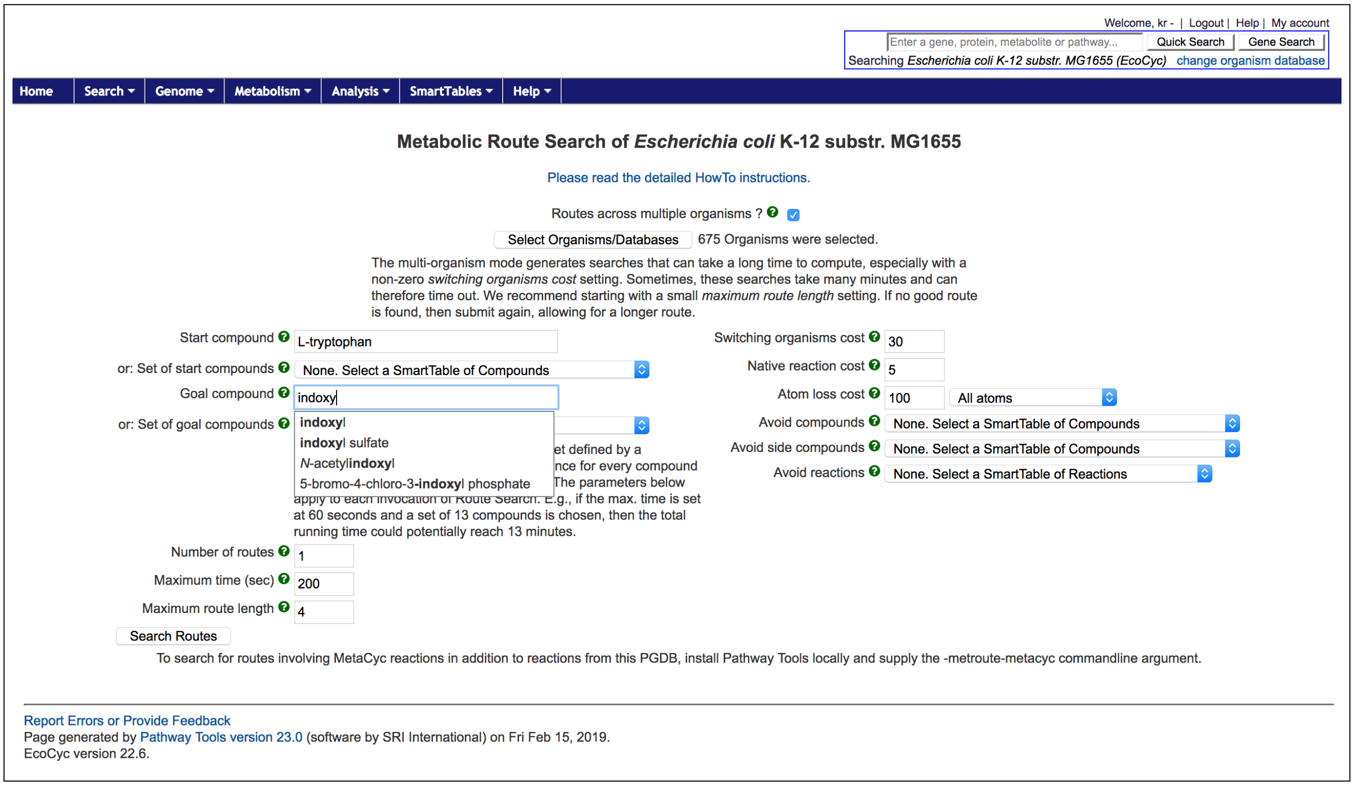

Figure 11: Likewise, enter the goal metabolite **indoxyl sulfate** in the same type of text box with completion, as was described above for the start metabolite.

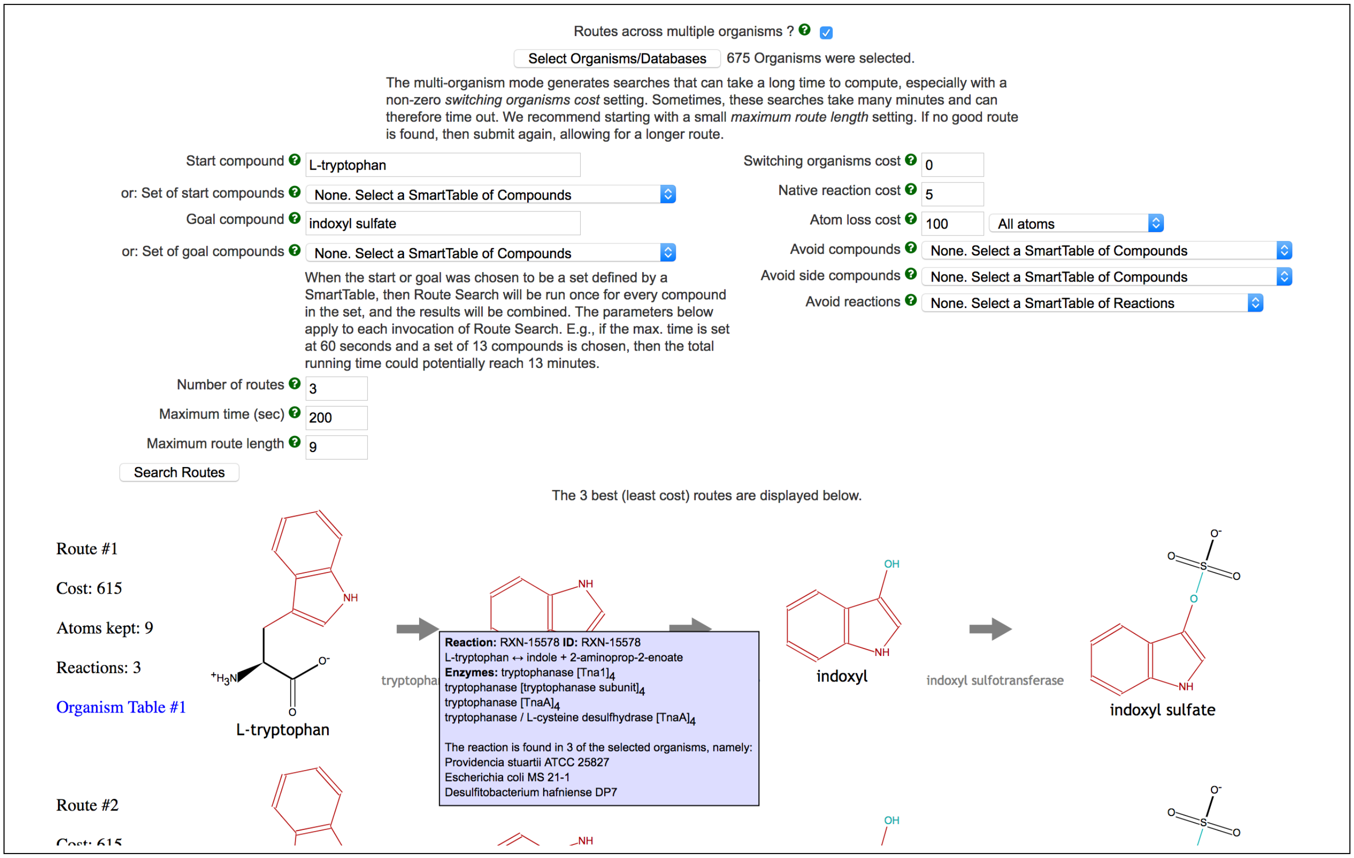

Figure 12: Set the **Number of Routes** to “3” and the **Maximum route length** to “9”. Note that this example also has the **Switching organisms cost** set to “0” (zero), to keep the running time fairly short. After all parameters for the search have been entered, as the last step, click on the **Search Routes** button. This launches the search, which generally could take anywhere between seconds to several minutes. A hard server timeout of 15 minutes will terminate any search, to reduce the load of BioCyc servers regarding ill-constrained searches. However, most Web browsers have their own timeout, which is set to only a few minutes. After the search is done, the resulting routes are displayed underneath. The thick gray arrows denote reactions between the primary metabolites. If the cursor is moved over such an arrow, a tooltip popup is displayed, containing information about the reaction, such as the EC classification number, the full reaction equation, names of enzymes catalyzing the reactions, and a count of organisms that could catalyze the reaction. In these graphical visualizations of metabolic routes, the retained atoms are depicted by coloring the conserved molecular fragments with the same colors. In this route, the main fragment is red. In other routes, several colors can be used for fragments that persist over a few reaction steps, but which do not persist through the entire route.

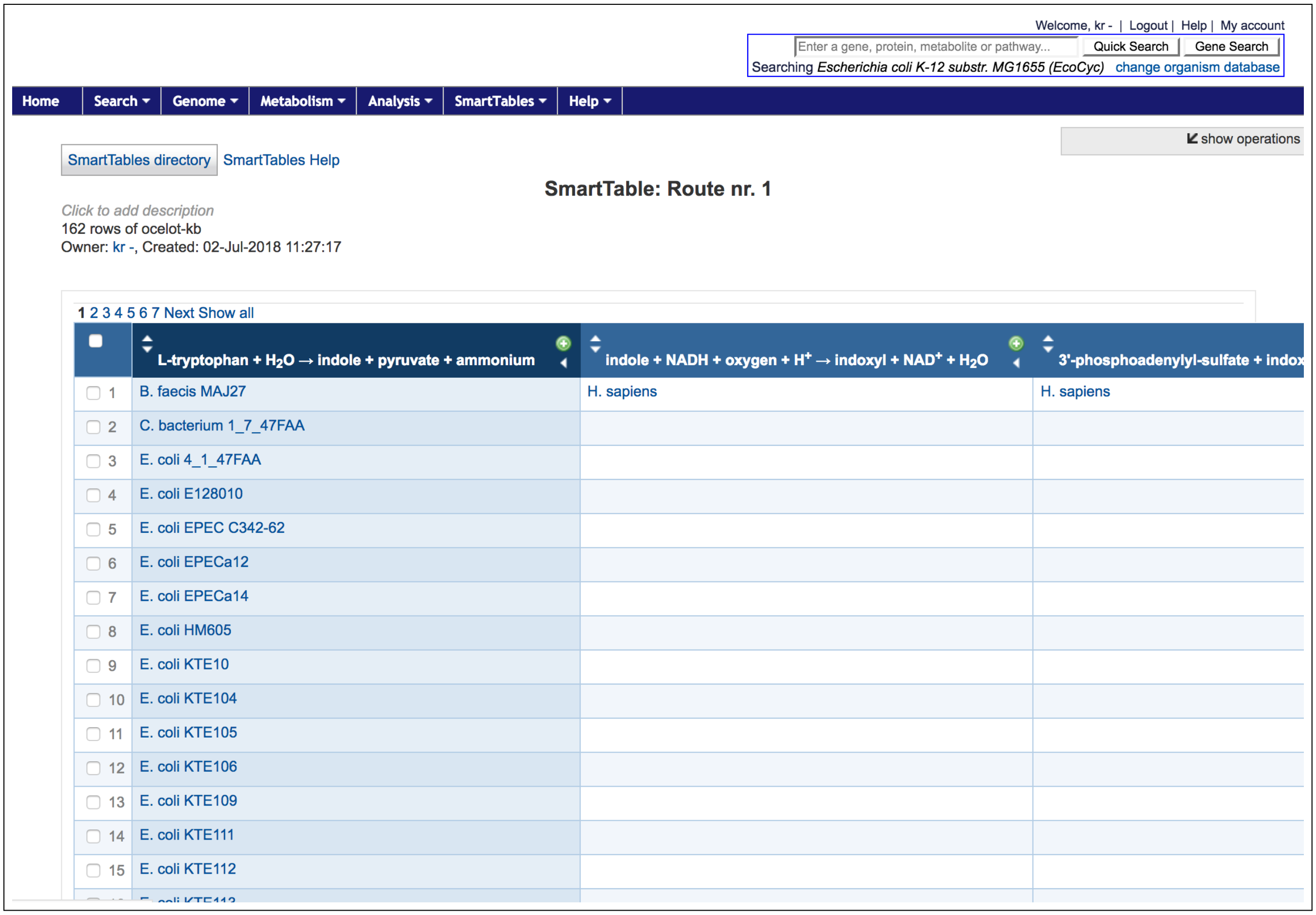

Figure 13: At the left side of each route, clicking a link called "Organism Table" will open a new SmartTable (SMT) in a browser tab. The browser's popup blocker may have to be advised to allow creation of a new tab, and the researcher needs to be logged into their BioCyc user account. This table shows the reactions of the route as its columns, and in each column, the rows list the organisms containing the reaction, in alphabetical order. This table is exportable in a Tab-delimited spreadsheet format, for further data analysis. To simplify external analysis, the SMT contains the organism results twice, by repeating the reaction columns and thus showing the route twice. The left side shows human-readable organism names, whereas the repeat on the right side of the table, separated by a blank separator column, records the unique organism IDs, which are easier to computationally process. When organism switching minimization is active, i.e. the "Switching organisms cost" parameter is non-zero, then the positions in a route, where the switches occur, are indicated by an inserted blank column. Additionally, the sorting of the organisms in table columns differs in the following way. We want to emphasize the organisms that can contain the longest stretches of the route, entirely within those organisms. So all the organisms are placed at the top, which can catalyze the entire stretch of reactions between organism switches, such that this property becomes visibly clear. Underneath these top organisms, other organisms will be listed that may only participate in shorter reaction stretches. Because the number of organisms involved per reaction becomes smaller as we move towards the right, the sorting has to be done from the right to the left, such that the few organisms that manage to catalyze the full stretch can determine the organisms to be shown at the top. Even though there might be more than one series of maximal sets of organisms with a minimum number of switching of organisms in a route, our current algorithm provides and displays only one of these series.
