# Supplementary figures and images for: Metabolic route computation in organism communities
Source: Microbiome. 2019 Jun 7;7:89. doi: 10.1186/s40168-019-0706-6 (PMC6556054; doi:10.1186/s40168-019-0706-6)

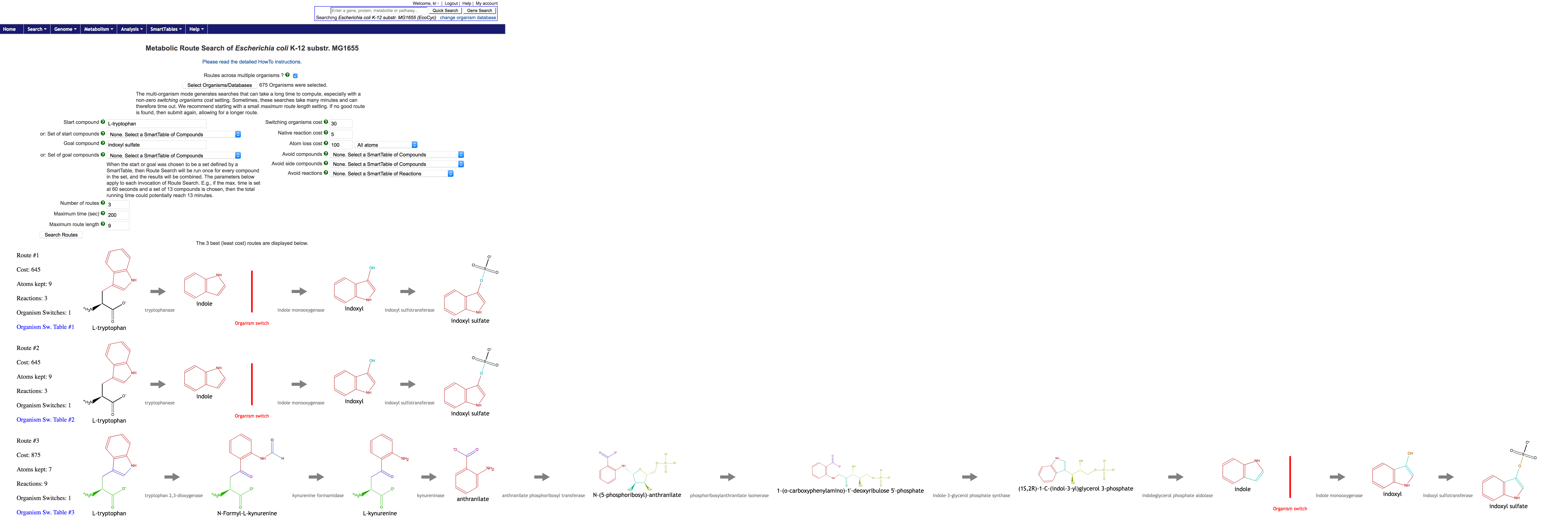

Supplement: Supplementary file 3 — L-tryptophan to Indoxyl Sulfate, two routes. The MORS interface controls and their selections are shown, followed by the resulting two routes computed by MORS. (PNG 756 kb) [file 40168_2019_706_MOESM3_ESM.png]

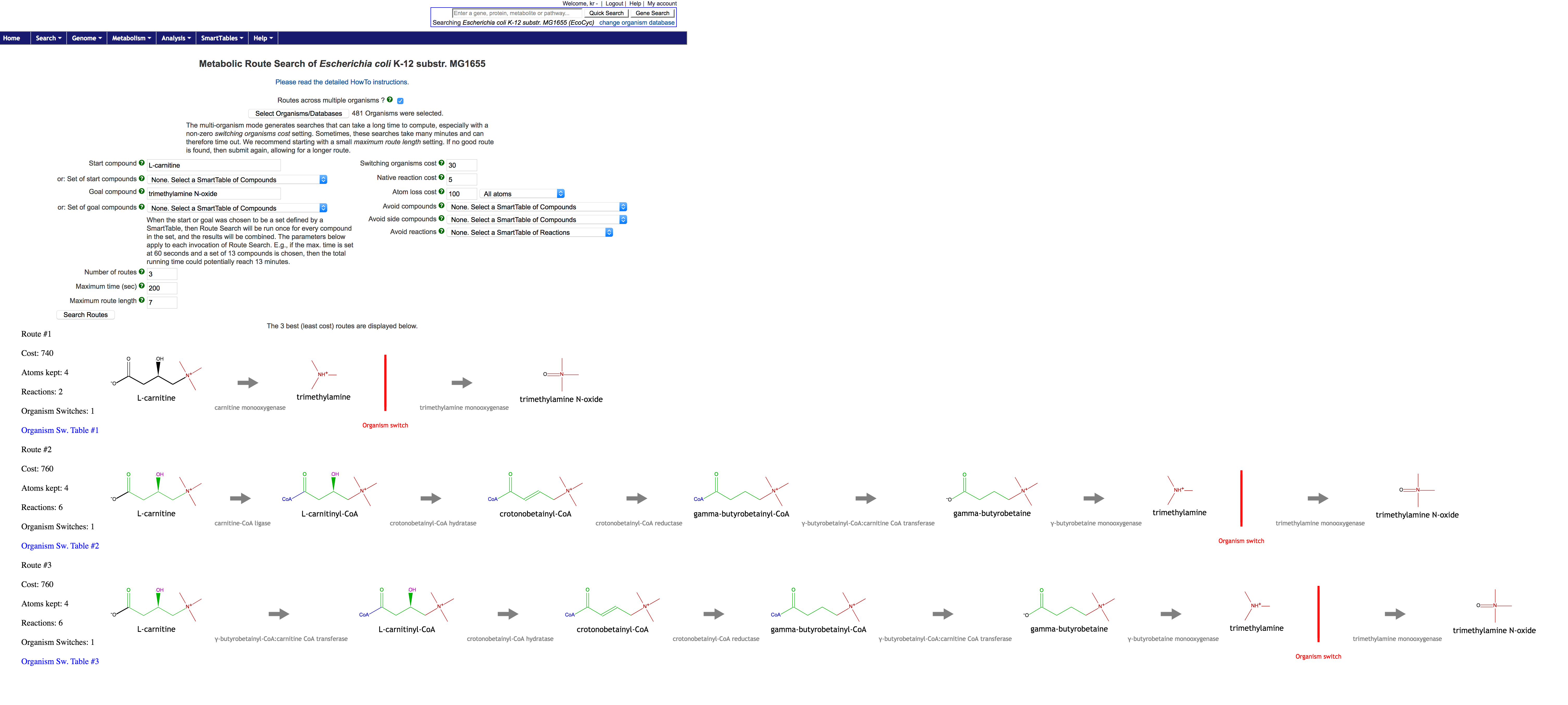

Supplement: Supplementary file 4 — L-carnitine to TMAO, three routes. The MORS interface controls and their selections are shown, followed by the resulting three routes computed by MORS. (PNG 594 kb) [file 40168_2019_706_MOESM4_ESM.png]
